# Identification of ADGRE5 as discriminating MYC target between Burkitt lymphoma and diffuse large B-cell lymphoma

**DOI:** 10.1186/s12885-019-5537-0

**Published:** 2019-04-05

**Authors:** Karsten Kleo, Lora Dimitrova, Elisabeth Oker, Nancy Tomaszewski, Erika Berg, Franziska Taruttis, Julia C. Engelmann, Philipp Schwarzfischer, Jörg Reinders, Rainer Spang, Wolfram Gronwald, Peter J. Oefner, Michael Hummel

**Affiliations:** 1 Charité – Universitätsmedizin Berlin, corporate member of Freie Universität Berlin, Humboldt-Universität zu Berlin, and Berlin Institute of Health, Institute of Pathology, D-10117 Berlin, Germany; 2 Statistical Bioinformatics, Institute of Functional Genomics, University of Regensburg, D-93053 Regensburg, Germany; 3 Functional Genomics, Institute of Functional Genomics, University of Regensburg, D-93053 Regensburg, Germany; 4 Present address: Department of Marine Microbiology and Biogeochemistry, NIOZ Royal Netherlands Institute for Sea Research, 1790 AB Den Burg, The Netherlands

**Keywords:** ADGRE5, CD97, MYC, ChIP-Seq, RNA-Seq, Lymphoma, BL, DLBCL

## Abstract

**Background:**

MYC is a heterogeneously expressed transcription factor that plays a multifunctional role in many biological processes such as cell proliferation and differentiation. It is also associated with many types of cancer including the malignant lymphomas. There are two types of aggressive B-cell lymphoma, namely Burkitt lymphoma (BL) and a subgroup of diffuse large cell lymphoma (DLBCL), which both carry *MYC* translocations and overexpress MYC but both differ significantly in their clinical outcome. In DLBCL, *MYC* translocations are associated with an aggressive behavior and poor outcome, whereas MYC-positive BL show a superior outcome.

**Methods:**

To shed light on this phenomenon, we investigated the different modes of actions of MYC in aggressive B-cell lymphoma cell lines subdivided into three groups: (i) MYC-positive BL, (ii) DLBCL with MYC translocation (DLBCLpos) and (iii) DLBCL without MYC translocation (DLBCLneg) for control. In order to identify genome-wide MYC-DNA binding sites a chromatin immunoprecipitation followed by high-throughput sequencing (ChIP-Seq) was performed. In addition, ChIP-Seq for H3K4me3 was used for determination of genomic regions accessible for transcriptional activity. These data were supplemented with gene expression data derived from RNA-Seq.

**Results:**

Bioinformatics integration of all data sets revealed different MYC-binding patterns and transcriptional profiles in MYC-positive BL and DLBCL cell lines indicating different functional roles of MYC for gene regulation in aggressive B-cell lymphomas. Based on this multi-omics analysis we identified *ADGRE5* (alias *CD97*) - a member of the EGF-TM7 subfamily of adhesion G protein-coupled receptors - as a MYC target gene, which is specifically expressed in BL but not in DLBCL regardless of *MYC* translocation.

**Conclusion:**

Our study describes a diverse genome-wide MYC-DNA binding pattern in BL and DLBCL cell lines with and without MYC translocations. Furthermore, we identified ADREG5 as a MYC target gene able to discriminate between BL and DLBCL irrespectively of the presence of *MYC* breaks in DLBCL. Since ADGRE5 plays an important role in tumor cell formation, metastasis and invasion, it might also be instrumental to better understand the different pathobiology of BL and DLBCL and help to explain discrepant clinical characteristics of BL and DLBCL.

**Electronic supplementary material:**

The online version of this article (10.1186/s12885-019-5537-0) contains supplementary material, which is available to authorized users.

## Background

The transcription factor MYC plays a multifunctional role in many cellular processes such as cell cycle progression, apoptosis and cellular transformation. Over-expression of MYC leads to an increased replication activity and is associated with different types of cancer. This holds also true for tumors of the immune system especially aggressive B-cell non-Hodgkin lymphomas (B-NHL) such as Burkitt lymphoma (BL) and diffuse large B-cell lymphoma (DLBCL). BL is an extremely fast growing tumor that carries immunoglobulin/*MYC* translocations in almost all cases. The tumor is predominantly found in male children but may also occur in adults especially with a compromised immune system. Treatment of BL is mainly based on high dose chemotherapy with usually favorable clinical outcome [1]. In contrast, DLBCL rarely carries *MYC* rearrangements, which may be associated with both immunoglobulin and non-immunoglobulin genes. Whereas DLBCL without *MYC* translocation reveals long-term survival of 60–70% of the patients treated with combined immune-chemotherapy, DLBCL with *MYC* translocation – regardless of its translocation partner – shows a very poor clinical outcome [2–8]. It is currently unclear why BL and DLBCL with *MYC* translocations display this very different clinical course. In addition, molecular features for a precise stratification of patients into BL and DLBCL with *MYC* translocation are lacking despite the need for different treatment modalities. To determine the potentially different role of MYC in BL and DLBCL, we aimed at identifying their molecular features by means of chromatin immunoprecipitation combined with high-throughput sequencing (ChIP-Seq) and whole transcriptome shotgun sequencing (RNA-Seq) employing B-cell lymphoma cell lines. Validation of the results was performed with primary lymphoma tissue samples.

## Methods

### Cell culture

**Fig. 1:**
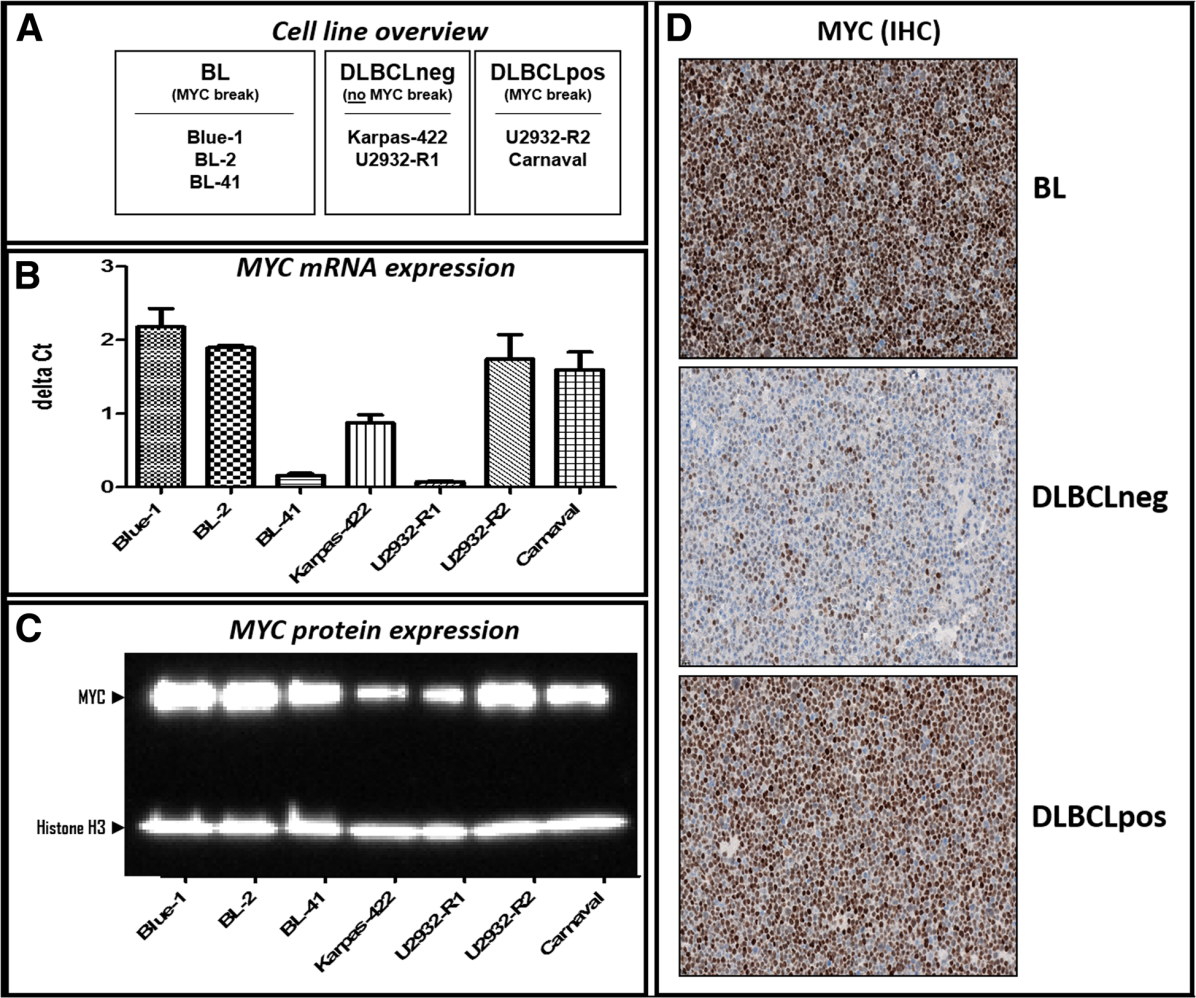
MYC expression in DLBCL and BL cell lines. **a** Cell lines categorized according to their genomic *MYC* status (*MYC* break). **b** Quantitative *MYC* RNA expression as determined by RT-PCR; endogenous control for normalization: B2M expression. **c** Western Blot analysis of MYC protein expression. **d** Immunohistochemical (IHC) staining for the cellular localization and distribution of MYC protein

Three *MYC* break positive BL cell lines (Blue-1 / ACC-594; BL-2 / ACC-625 and BL-41 / ACC-160), two *MYC* break positive (Carnaval / ACC-724; U2932-R2 / ACC-633) and two *MYC* break negative (Karpas-422 ACC-32, U2932-R1 / ACC-633) DLBCL cell lines (overview Fig. 1a) were obtained in 2012 from the German Collection of Microorganisms and Cell Cultures (DSMZ). The sub-clones U2932-R1 and U2932-R2 were kindly provided by Dr. Quentmeier (DSMZ, Braunschweig, Germany) [9]. All cell lines were negatively tested for mycoplasma contamination prior to use and are currently not listed as cross-contaminated or misidentified cell lines according the International Cell Line Authentication Committee (ICLAC). All cell lines were cultivated in RPMI 1640 medium supplemented with GlutaMAX™-I (Gibco, Thermo Fisher Scientific) and containing 20% of heat inactivated fetal bovine serum (PAN Biotech, Aidenbach, Germany) under a humidified atmosphere with 5% CO_2_ at 37 °C. Cells were thawed and continuously split 3 times per week for a maximum period of three weeks. Cell counting was performed on a BD Accuri C6 Flow Cytometer (BD Biosciences, New Jersey, *United States*) and cell viability was determined by propidium iodide (PI) – staining (BD Bioscience, Heidelberg, Germany) according to the manufacturer’s recommendations. Only cells, which exhibited more than 90% vitality, were used for further investigation.

### Western blotting

1.5 × 10^6^ vital cells were washed three times with PBS and lysed with protease inhibitors containing RIPA buffer supported by sonication. After measuring protein concentration using the BCA protein assay kit (Pierce, Thermo Fisher Scientific), protein lysates were separated under denaturing conditions via gels electrophoresis using 16% sodium dodecyl sulfate polyacrylamide gels (Invitrogen, California, *United States*) and transferred to Hybond-ECL nitrocellulose membranes (Amersham Biosciences, New Jersey, *United States*) by electroblotting. Membranes were blocked with a PBST 5% dry milk solution for 1 h followed by incubation with the respective primary antibody solution at 4 °C overnight. Subsequently, membranes were washed three times with PBST and incubated for 1 h with a secondary antibody conjugated with horseradish peroxidase (information on primary and secondary antibodies is available in Additional file 1: Table S1). Chemiluminescence was detected using HRP substrate (Luminata Forte, Merck Chemicals GmbH, Darmstadt, Germany) and FusionCapt Advance analysis Software (Fusion device, Vilber Lurmat GmBH, Eberhardzell, Germany).

### Quantitative real-time PCR analysis

Total RNA was isolated from 1 × 10^6^ vital cells after washing with PBS employing NucleoSpin RNA Kit (MACHEREY-NAGEL GmbH & Co. KG, Düren, Germany). RNA fluorometric quantification was performed by means of the Qubit RNA quantification assay (Thermo Fisher Scientific). Total RNA was reverse transcribed into complementary DNA (cDNA) using TaqMan reverse transcription reagents. Real-time PCR analysis was realized using TaqMan Real-Time PCR Master Mix on a Step One Plus Real-Time PCR System (Thermo Fisher Scientific). All procedures were performed according to the manufacturer’s recommendations. RT-PCR Taq-Man probes are listed in Additional file 1: Table S1. Relative RNA expression was calculated according to the comparative Ct method [10] using the average expression based on triplicates of two biological replicates of each cell line. For endogenous control b2-microglobulin (B2M) or succinate dehydrogenase complex, subunit A (SDHA) were used.

### ChIP-Seq experiments

Chromatin immunoprecipitation (ChIP) was done according to published protocols [11, 12] with few modifications. Briefly, 2 × 10^7^ vital cells were fixed for 10 min at 4 °C in medium containing 1% formaldehyde. After blocking with 0.1 M glycine and washing four times with PBS, the cells were snap frozen and stored at − 80 °C. After thawing on ice each cell pellet was resuspended in 5 mL cold LB1 lysis buffer, incubated for 10 min at 4 °C and for further 10 min ambient temperature in 5 mL LB2 lysis buffer before being finally dissolved in 3 mL LB3 buffer. Sonication was performed for 45 min [three cycles of 15 min each at high power in pulsed mode (30 s on and 30 s off)] using titanium rods combined with a Bioruptor Sonicator (Diagenode, Seraing, Belgium). After addition of 300 μL 10% (vol/vol) Triton X-100 and centrifugation the supernatant was removed, 50 μL of which were stored as input DNA sample. 1.5 mL of the supernatant was incubated with 10 μg MYC antibody or 5 μg H3K4me3 antibody at 4 °C overnight. For ChIP antibody information, refer to Additional file 1: Table S1. For precipitation of DNA indirectly bound to the respective antibody, 30 μg Dynabeads coupled with Protein G (Thermo Fisher Scientific) were added for each μg antibody and incubated for 3 h at 4 °C. Subsequently, the beads were washed and the immunoprecipitated (IP) DNA was eluted. Finally, the eluate (input DNA and IP DNA) was reverse cross-linked overnight at 65 °C followed by digestion with RNase A and Proteinase K. The resulting DNA was phenol/chloroform extracted, precipitated and the DNA was resuspended in 30 μL 10 mM Tris·HCl, pH 8.0. DNA was subjected to fluorometric quantification by the Qubit DNA quantification assay (Thermo Fisher Scientific). Ten ng of chromatin-immunoprecipitated DNA sample were processed with NEBNext ChIP-Seq Library Prep Master Mix Set for Illumina library generation according to the manufacturer’s recommendations. All amplified libraries were analyzed with the DNA 1000 Kit on the 2100 Bioanalyzer (Agilent, California, *United States*). Single-read NGS was done on an Illumina HiSeq 1500 system (50 cycles). Illumina adapters were trimmed from the raw sequence data and low quality bases and reads were removed with trimmomatic (LEADING:3 TRAILING:3 SLIDINGWINDOW:4:15 MINLEN:36) [13]. Sequence data was aligned to the main chromosomes of the human reference genome (GRCh38) with bowtie version 0.12.7 (−e 70 -k 1 -m 1 -n 2 –best) [14]. H3K4 and *MYC* peaks were called with MACS2 [15] with a q-value cut-off of 0.1 and the peaks from the two replicate ChIP samples were summarized with IDR [16], keeping all peaks with an IDR < 0.1. Final peaks were annotated to the nearest transcription start site (TSS) using gene annotation from Ensembl release 77. Only peaks with a maximum distance of 2000 bp to a TSS were kept. Artificial peaks were removed using the ENCODE blacklist (https://sites.google.com/site/anshulkundaje/projects/blacklists). Differential peaks between DLBCL with and without *MYC* break and BL were estimated using DiffBind [17] tool.

### RNA-Seq analysis

Total RNA was isolated from 1 × 10^6^ lymphoma cells, which were previously spiked in with 1 × 10^5^ insect cells (Schneider cells) for data calibration [18]. The quality of the RNA was determined with an Agilent 4200 TapeStation and Software A.01.05 (Agilent, California, *United States*). 500 ng RNA per sample were processed using the Illumina TruSeq Stranded mRNA LT Sample Prep Kit following the manufacturer’s instructions to generate libraries for RNA sequencing. Samples were sequenced on a Hi-Seq 4000 (single read mode; length 150 bp) using the Illumina HiSeq 3000/4000 SBS 150 cycle kit. Sequence reads were aligned to a concatenated genome that consisted of the human (GRCh38) and the *Drosophila melanogaster* (BDGP5) reference genome, using STAR alignment tool [19] with default parameters. Gene annotation from Ensembl release 77 and feature Counts [20] with default parameters were used to assign read counts to human and *Drosophila* genes. Before differential gene expression analysis, we calculated DESeq2 sample sizeFactors [21] on the *Drosophila* gene counts and applied them to the human sample data. This way, gene expression levels of the cell lines were calibrated to the number of sample cells. Then, gene expression levels were modeled with a generalized linear model assuming negative binomial distributed data and categorical variables for the lymphoma type (BL or DLBCL) and *MYC* status (*MYC* break positive or negative). Gene expression changes were tested for significance with the Wald test and fold changes with an associated False Discovery Rate (FDR) below 0.05 were considered significant differentially expressed.

### Proteomics

The SWATH-MS-based quantification of the proteins ADGRE5, BYSL and NPM1 was obtained from previously published data [22]. SWATH-MS measurements were carried out on a TripleTOF 5600+ (Sciex, Darmstadt, Germany) coupled to an Ultimate 3000 nano-HPLC-system (Dionex, Idstein, Germany) using an 88 min-binary gradient. The PeakView 2.1 software (Sciex, Darmstadt, Germany) was employed for quantification of the peptides based on an in-house library. Only peptides with FDR < 1% and confidence > 95% were considered for quantification. Peptide intensities were summed up and normalized to total protein intensity. Statistical tests were conducted using heteroskedastic 1-way ANOVA.

### Immunohistochemistry

Immunohistochemical staining was performed using sections derived from formalin-fixed paraffin-embedded cell line blocks (*n* = 12) and primary tissue samples (*n* = 38). The use of human primary tissue samples was approved by the Institutional Review Board of the Charité – Berlin (EA4/104/11). The immunostaining carried out using the Leica Bond-maX autostainer (Leica Biosystems, Illinois, *United States*) according to the manufacturer’s protocol. After heat-induced epitope retrieval, the sections were incubated with anti-c-myc and anti-CD97 (ADGRE5) rabbit antibodies, respectively (dilution 1:200). Horseradish peroxidase-labeled Anti-rabbit-IgG using the Bond Polymer Refine Detection Kit (Leica Biosystems, Illinois, *United States*) was employed to convert the chromogen substrate. Staining was performed with appropriate positive and negative controls.

## Results

First, we determined *MYC* mRNA and MYC protein expression by qRT-PCR, Western blotting and immunohistochemistry, respectively, in cell lines derived from BL, DLBCLpos and DLBCLneg patients (Fig. 1 b-d). With the exception of BL-41, all *MYC* break positive cell lines showed high expression of *MYC* mRNA. The level of MYC protein expression corresponded without exception with the presence of *MYC* breaks. The discrepant results between *MYC* RNA and MYC protein expression in BL-41 might reflect a longer half-life time of the MYC protein in BL-41 as compared to the other cell lines with *MYC* breaks [23–27]. Thus, less RNA is required to generate high amounts of MYC protein.

To investigate the MYC DNA-binding capabilities in BL and DLBCL, we performed MYC ChIP-Seq experiments to determine genome-wide MYC DNA-binding sites. Additional ChIP-Seq experiments for trimethylation of histone H3 at lysine 4 (H3K4me3) were carried out in order to locate genomic areas with open chromatin as indicators for potential transcriptional activity of nearby genes [28, 29]. To bioinformatically identify differential MYC DNA-binding sites the DiffBind package [17] was employed using a pairwise comparison of the cell groups (BL vs. DLBCL; BL vs. DLBCLpos; BL vs. DLBCLneg and DLBCLpos vs. DLBCLneg). Similar differential binding analysis was performed with H3K4me3 ChIP-Seq data to ascertain genome wide differential histone patterns and potential active transcriptional sites.

**Fig. 2:**
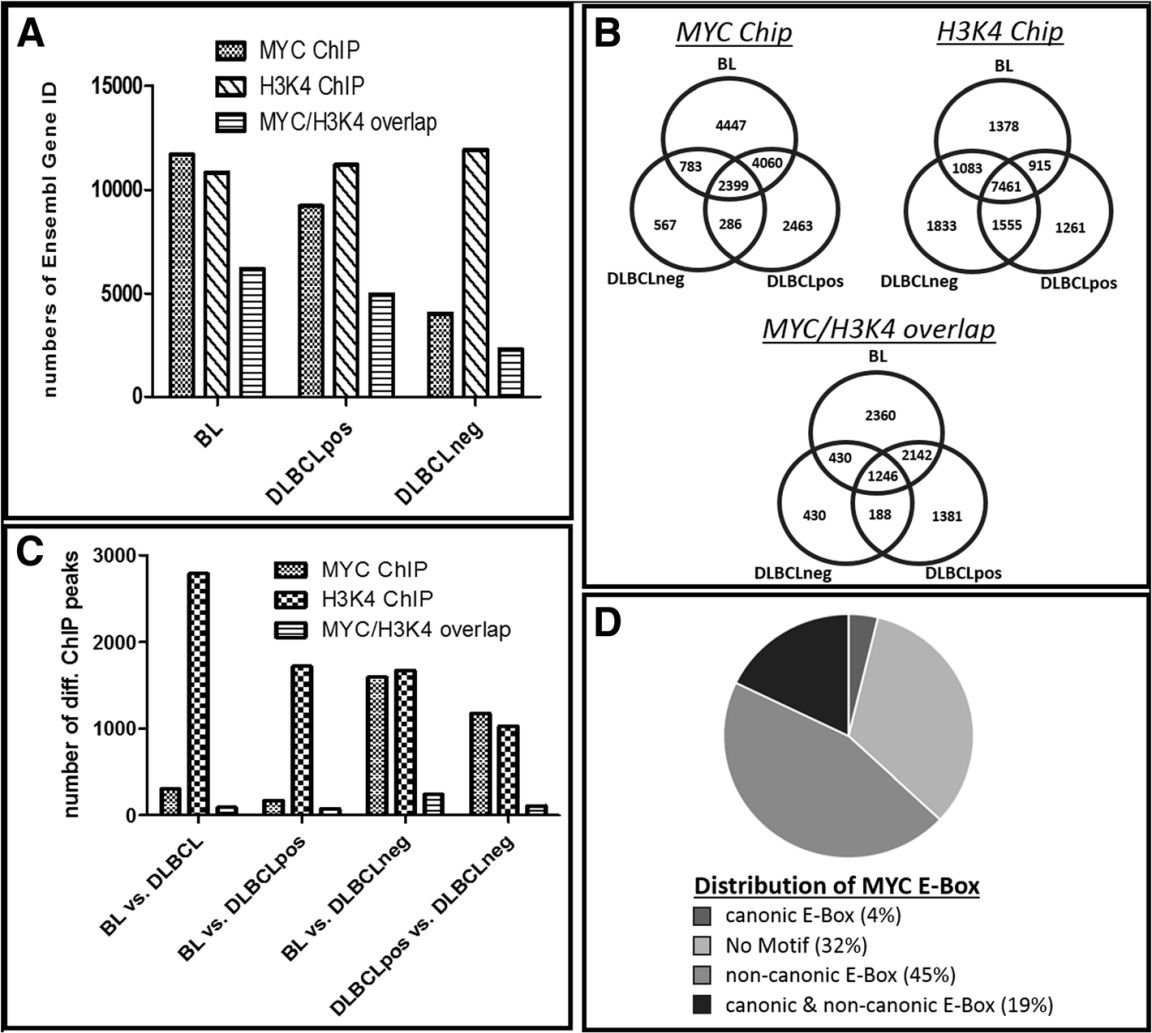
Differential binding patterns obtained by ChIP-Seq experiments. **a** Total gene counts identified by MYC-Chip, H3K4me3-ChIP, and an overlay of MYC/H3K4me3-ChIP peaks after MACS2 IDR peak calling. **b** Venn diagrams illustrate the number of identified targets after IDR peak calling of MYC and H3K4 ChIP, respectively, limited to within 2000 bp from Origin of Replication (ORI). Each count presents a single Ensembl gene ID. **c** Differential binding analysis between different lymphoma entities. Each count presents a single Ensembl gene ID, limitation by 2000 bp of ORI, IDR < 0.1 and *p*-value < 0.05. **d** Distribution of MYC E-Box binding motif within the identified genes with differential MYC-Chip peaks

**Fig. 3:**
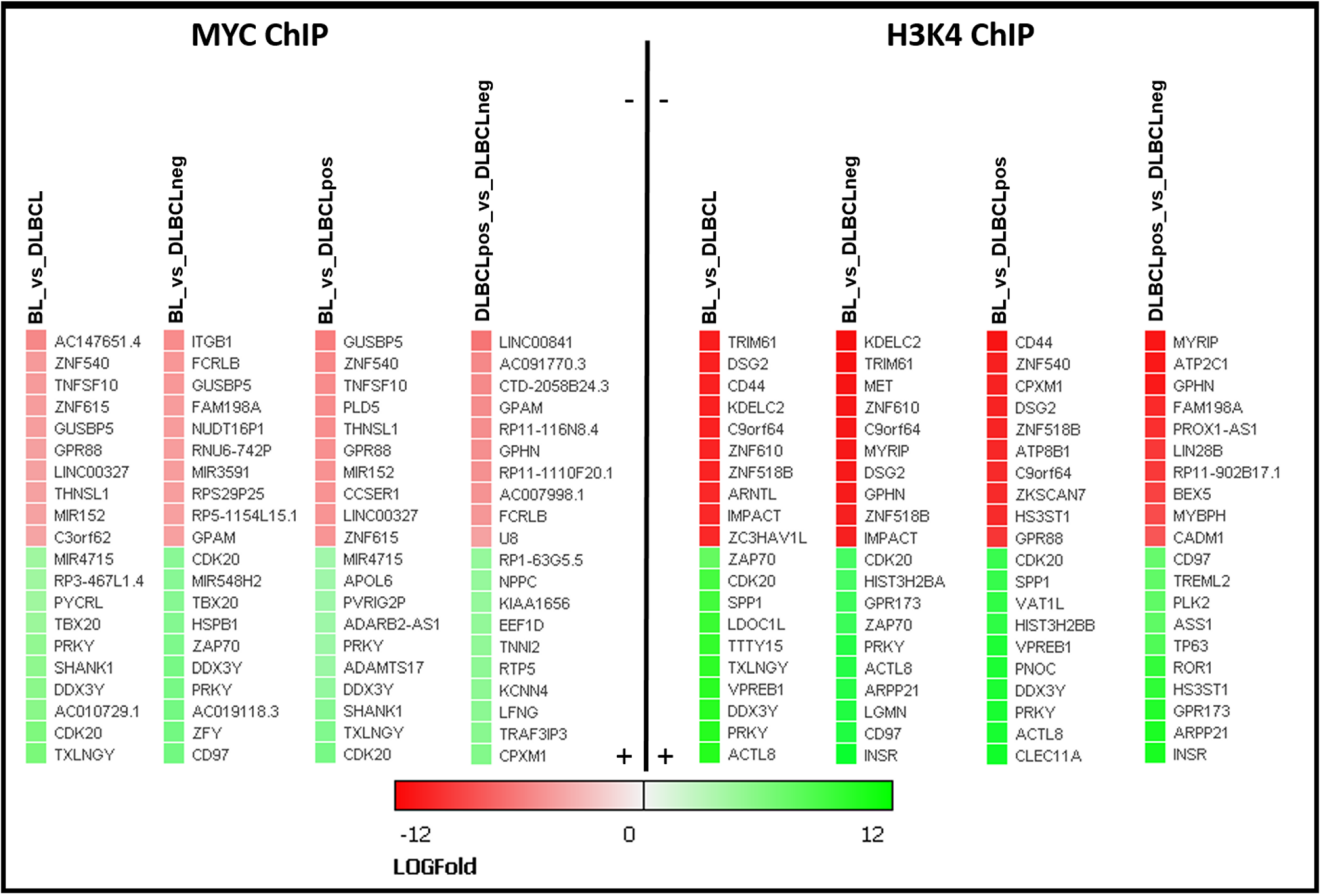
Selected differentially bound genes derived from MYC and H3K4me3 ChIP-Seq experiments. Twenty selected target genes with a highest Log Fold Change value obtained from differential binding analysis for each pairwise comparison (BL_vs_DLBCL; BL_vs_DLBCLneg; BL_vs_DLBCLpos; DLBCLpos_vs_DLBCLneg). Restriction to 2000 bp upstream of transcriptional start site; IDR < 0.1 and *p*-value < 0.05

Detailed results of the bioinformatics analyses are available in Additional file 2: File S2(ChIP-Seq data), while Figs. 2 and 3 depict aggregated data. The overall number of MYC DNA-binding sites was higher (approx. 2-fold) in *MYC* break positive (BL, DLBCLpos) than *MYC* break negative (DLBCLneg) cells (Fig. 2a). Next, we explored whether genes associated with MYC-binding differed between the three groups of cell lines. Our data clearly indicate that there is not only a difference in the number of genes but in addition, that also different genes are targeted by MYC and/or H3K4 (Fig. 2b). To identify differential MYC-binding genes we performed a differential peak analysis comparing four combinations: BL vs. DLBCL, BL vs. DLBCLneg, DLBCLpos vs. DLBCLneg and BL vs. DLBCLpos. Cell lines carrying *MYC* breaks have more genes located in the vicinity of MYC-binding sites which leads to a higher number of differential MYC-binding peaks in relation to *MYC* break negative cell lines (Fig. 2c). Figure 3 highlights a list of twenty target genes selected that yielded the highest fold changes. The analysis of the MYC-binding motifs of MYC target genes showed an interesting distribution (Fig. 2d) with a preference for non-canonical E-Box motives (approx. 45%), while only 4% carried exclusively the classical canonic E-Box motif (CACGTG) and 19% both motifs. Strikingly, 32% of identified MYC targets genes displayed no known MYC-binding motifs. Non-canonical and/or canonical E-box was present in approx. 68% of MYC target genes, thus corroborating previous studies of global mapping of MYC-binding sites [30]. However, the presence of E-box motives in the binding loci did not correlate with the regulation of associated genes [31, 32].

**Fig. 4:**
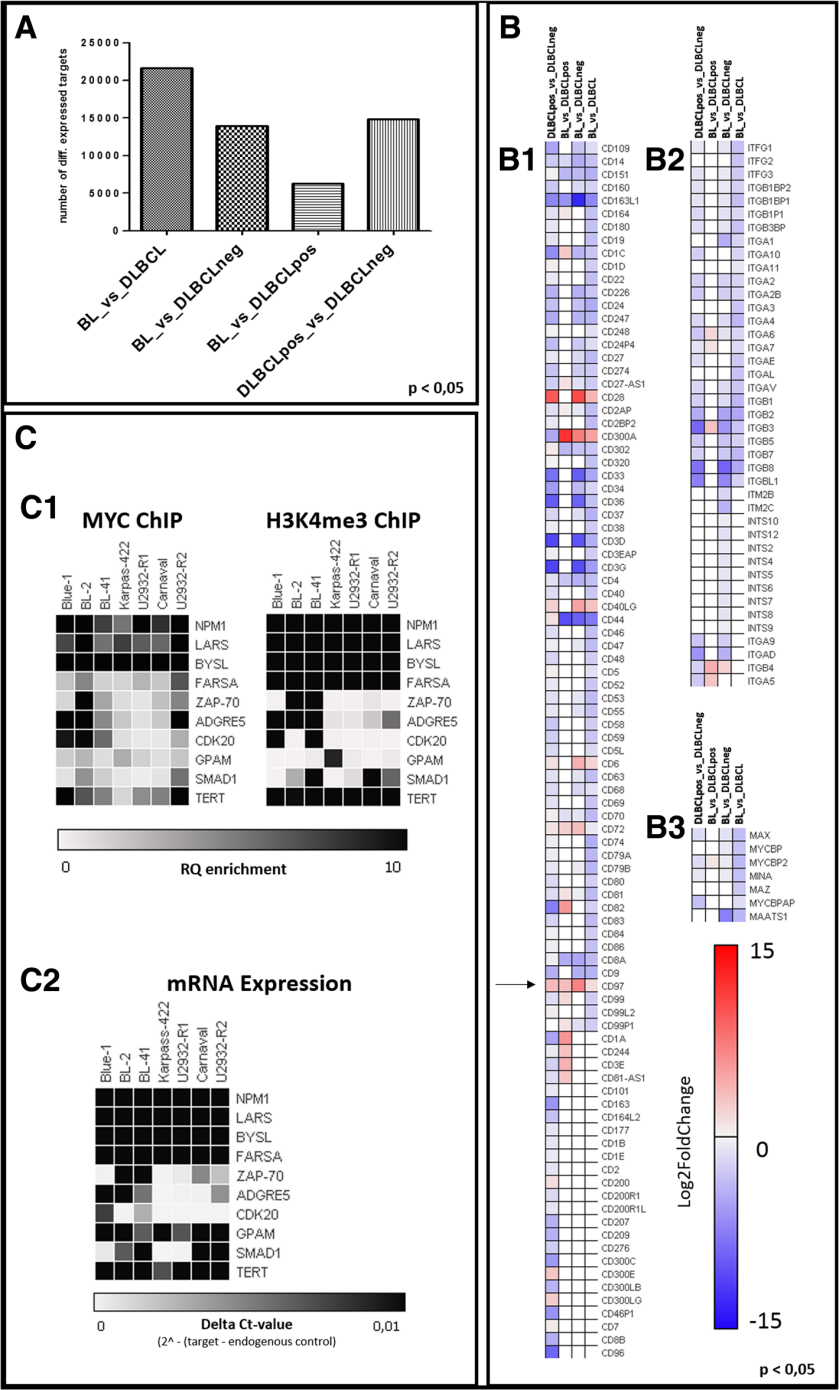
RNA-Seq and validation of selected targets. **a** Overview of total counts of identified RNA-Seq targets after differential expression analysis between pairs of lymphoma (sub-) entities. **b** Exemplary heatmaps of differentially expressed targets grouped for (B1) clustering of differentiation molecules, (B2) integrin molecules, and (B3) MYC-related molecules. **c** Summary of the validation experiment for selected targets via additional MYC/H3K4me3 ChIP enrichment analysis (C1) and additional TAQ-MAN RT-PCR analyses (C2) in BL (Blue-1; BL-2, BL-41), DLBCLneg (Karpas-422; U2932-R1) and DLBCLpos (U2932-R2; Carnaval) cell lines (*n* = 2 biological replicates)

MYC-binding is not the sole factor for activating of gene expression and associated functional consequences. In order to gain a deeper insight into the transcriptional landscape and the impact of the various MYC-binding patterns, we performed RNA-Seq and correlated the results with the presence of *MYC* breaks and with the MYC and H3K4 binding patterns. In Fig. 4a the number of genes differentially expressed among the three cell line groups is given. The highest number of differentially expressed genes was found between BL and DLBCL in general, whereby the expression difference between BL and DLBCLpos was the lowest. This demonstrates that MYC has a major impact through activation of the same gene set, which constitutes a significant proportion of the entire transcriptome. In harmony with this notion, the comparison of the RNA-Seq data between BL and DLBCLneg, and DLBCLpos and DLBCLneg revealed very similar numbers of differentially expressed genes. This reinforces the similarity in the gene expression profiles of both types of *MYC* break positive cell lines. Lists of differentially expressed genes are given in Additional file 3: File S3(RNA-Seq data). In Fig. 4b some differentially expressed genes (from Additional file 3: File S3) are functionally grouped into clustering of differentiation (CD) molecules (B1), integrin molecules (B2) or MYC-related molecules (B3) and visualized as heat maps. Most of the identified CD molecules seem to be upregulated in *MYC* break positive (BL, DLBCLpos) cell lines compared to *MYC* break negative (DLBCLneg) cell lines.

To validate the data derived from genome-wide DNA-binding and gene expression, we performed additional gene-specific ChIP (MYC and H3K4me3) and RT-PCR experiments (Fig. 4c). The selection criteria for the target genes were MYC DNA-binding according to ChIP-Seq and differential expression according to RNA-Seq. Among the identified genes, *ZAP-70*, *ADGRE5*, *CDK20*, *GPAM*, *SMAD1* and *TERT* were the most interesting. Genes lacking differentially expression such as *LARS, FARSA* and already described as MYC target genes like *BYSL* [33] and *NMP1* [34] were selected as positive control. By independent validation assays we were able to confirm the data derived from our ChIP-Seq and RNA-Seq analyses and demonstrate that a set of genes is able to reliably differentiate between lymphoma entities.

Interestingly *ADGRE5* (previously designated as *CD97* and marked by an arrow in Fig. 4, B1) shows strong selective expression in BL cell lines. On the other hand, some integrin molecules known to be binding partners of ADGRE5 are downregulated in BL as compared to DLBCL.

**Fig. 5:**
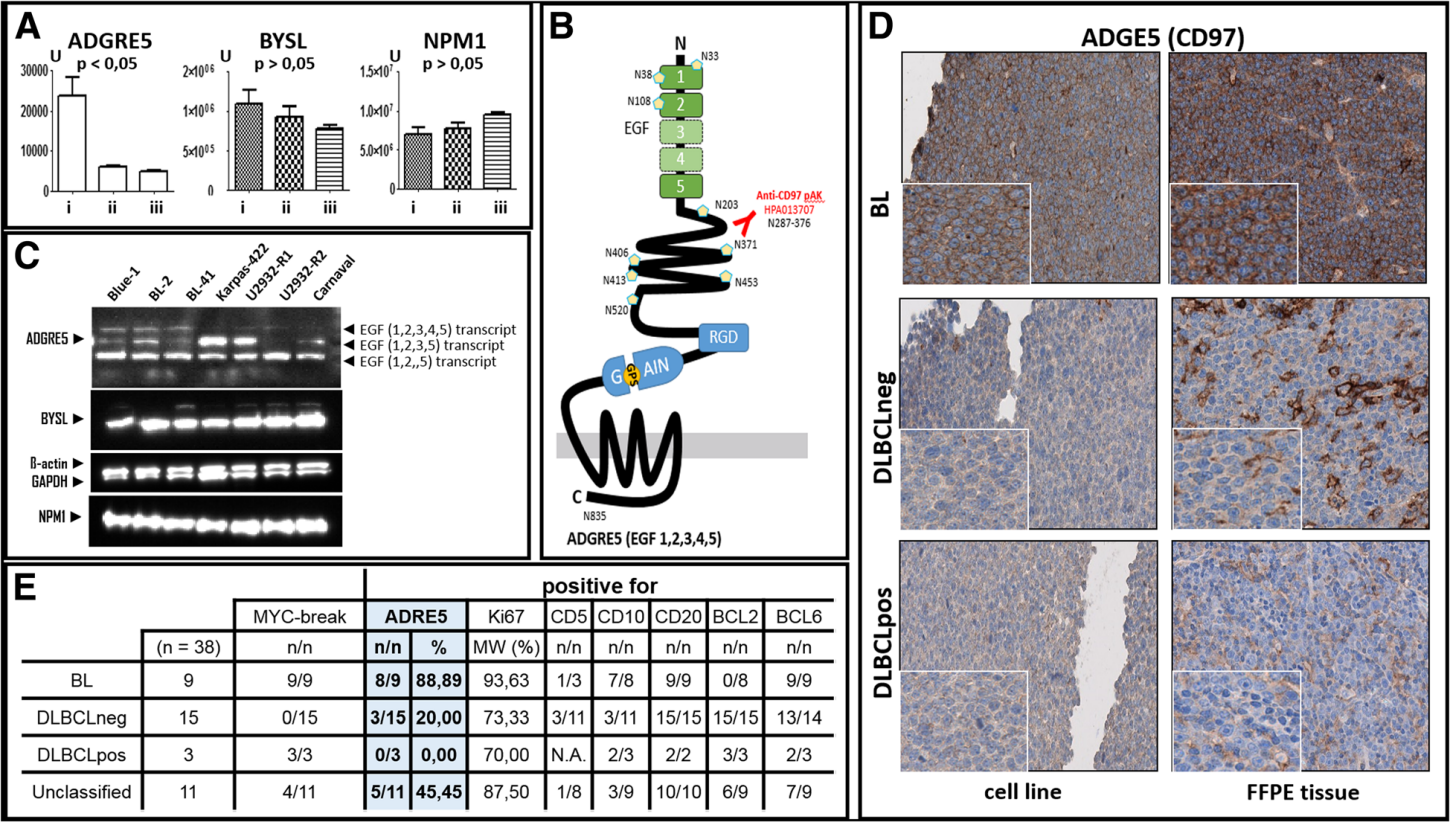
ADGRE5 (alias CD97) protein expression in cell lines and patient tumor samples. **a** Proteomic analysis of ADGRE5, BYSL and NPM1 level in BL (i), DLBCLneg (ii) and DLBCLpos (iii) cell lines. **b** Schematic model of largest ADGRE5 isoform EGF (1,2,3,4,5), Arg-Gly-Asp integrin-binding motif (RGD), GPCR-autoproteolysis-inducing domain (GAIN), epidermal growth factor domain (EGF), and nucleoside position of potential N-glycosylation sites are indicated. **c** Western Blot analysis of expression of ADGRE5, BYSL, NMP1 and endogenous control ß-actin and GAPDH in selected cell lines. **d** Immunohistochemical (IHC) staining of ADGRE5 protein in BL, DLBCLpos and DLBCLneg cell lines and FFPE tumor tissue samples. **e** Overview of ADGRE5 staining FFPE tumor tissue samples and supplementary information

To validated this interesting outcome we quantify the proteins of ADGRE5 and already known homogeneous expressed MYC targets like BYSL and NPM1, obtained from previously published proteomic data [22] (Fig. 5 a) and western blot analysis (Fig. 5 c). Finally, we demonstrated the discriminating character of ADGRE5 between BL and DLBCL in additional immunostainings of cell lines and FFPE tissue samples (Fig. 5 d).

## Discussion

Our RNA-Seq and ChIP-Seq data showed a significant overrepresentation of ADGRE5 in BL as compared to DLBCL regardless of the *MYC* break status of the latter. This finding was confirmed by independent additional target-specific ChIP experiments, RT-PCR and re-analysis of published proteomic data [22] (see Figs. 4 C1-2 and 5 a). For further exploration, we selected *ADGRE5* as an interesting candidate gene. ADGRE5 previously designated as CD97 [35] belongs to the adhesion G protein-coupled receptor (GPCR) subfamily E and was the first receptor of this gene family found to be associated with cancer [36]. ADGRE5 is a member of the EGF-TM7 (seven-span transmembrane protein containing epidermal growth factor domains; Fig. 5 b) protein family and is constitutively expressed in granulocytes, monocytes as well as in subsets of T- and B-cells [37–42]. An increased ADGRE5 expression is found in some types of leukemia [43–46]. Interestingly, differential expression of *ADGRE5* has also been described for several solid cancers such as lung, thyroid and colorectal carcinomas, indicating a tumor and/or tissue-specific expression pattern [47–49]. Upregulation of ADGRE5 is often observed at the invading tumor front as well as in advanced tumor stages. Furthermore, ADGRE5 presents an unfavorable prognostic factor [50–54]. Depending on the cell type and tumor grade, ADGRE5 protein exists in three isoforms resulting from alternative splicing [55]. ADGRE5 protein is cleaved by self-catalytic proteolysis into a large extracellular subunit, which contains three (EGF1,2,5), four (EGF1,2,3,5) or five (EGF1,2,3,4,5) extracellular N-terminal epidermal growth factor (EGF)-like domains, that are coupled to the seven-span transmembrane subunit (TM7) via an extended spacer region [56–58]. As a surface receptor molecule, ADGRE5 has the ability to bind ligands of the cellular and extracellular matrix, enhances proteolytic activity of matrix metalloproteinases (MMPs) and thus triggers secretion of chemokines [59]. Finally, ADGRE5 interacts with CD55 [60–63], the glycosaminoglycan chondroitin sulfate [64, 65], integrin [66] or CD90 [67] in an isoform-specific manner. Initial functional studies suggest that ADGRE5 is relevant for cell adhesion, migration and invasion [53, 59, 68].

To determine ADGRE5 isotype distribution in aggressive lymphoma, we performed Western blot analyses (Fig. 5c) and found that the short isoform (EGF1,2,5) is homogeneously expressed in all cell lines, while the largest isoform (EGF1,2,3,4,5) was preferentially present in BL cell lines. This is a very striking finding, as the EGF-like repeat 4, which has been reported to interact with chondroitin sulfate, is only found in the largest ADGRE5 isoform [64–66]. The interaction of chondroitin sulfate and ADGRE5 (EGF4) mediates cell adhesion and angiogenesis and plays an important role in the interaction of activated T-cells, dendritic cells and macrophages. This observation fits very nicely to features of BL, especially angiogenesis and macrophage attraction.

In order to determine the expression profile of ADGRE5 in primary patient specimens, we performed immunohistochemical staining (IHC). Figure 5d illustrates the higher expression of ADGRE5 on the cell surface of BL cell lines and primary BL patient specimens. In contrast, primary tissue specimens obtained from DLBCL patients and cell lines largely lacks ADGRE5 protein expression irrespectively of the presence of *MYC* breaks. (Additional IHC staining results are shown in Additional file 4: Figure S4). Table 5E summarizes the ADGRE5 IHC results obtained for 38 patients suffering from BL and DLBCL, respectively. Overall, ADGRE5 is significantly more frequently positive in BL patients (88%) as compared to DLBCLs patients that were mainly negative (80%). Thus, the data derived from our cell line experiments are nicely reflected in primary patient specimens.

## Conclusion

Here we describe the impact of MYC in three types of aggressive B-cell non-Hodgkin lymphomas: BL and DLBCL with and without MYC break (DLBCLneg and DLBCLpos, respectively). More MYC-binding sites were found by MYC ChIP-Seq in BL and DLBCLpos as compared to DLBCLneg. Interestingly, MYC was found to be bound to different target genes in BL and DLBCLpos, which is also reflected by their gene expression differences. By combined analyses, ADGRE5 (CD97) was identified as an interesting differentially expressed MYC target gene, an observation confirmed by immunohistochemistry of primary FFPE patient samples. Its expression, in particular that of the largest ADGRE5 isoform (EGF1,2,3,4,5), was significantly higher in BL than DLBCL. Based on the reported function of the EGF4 repeat as a receptor for chondroitin sulfate, we hypothesize that this might contribute to some peculiar features of BL, namely macrophage attraction and angiogenesis, and potentially to the very good responsiveness to treatment. In addition, we suggest ADGRE5 as a marker to discriminate between BL and DLBCL (regardless of the presence of MYC breaks) in patient stratification.

## Additional files

Additional file 1: **Table S1.** Compilation of antibodies used for Chromatin Immunoprecipitation (ChIP), Western Blot (WB) or Immunohistochemistry (IHC) and list of TaqMan Assays used for RT-PCR analysis. *indicated endogenous control. (PDF 42 kb)

Additional file 2: **File S2.** ChIP-Seq data of differentially bound MYC and H3K4 target genes derived from comparison of BL vs. DLBCL, BL vs. DLBCLneg, DLBCLneg vs. DLBCLpos and BL vs. DLBCLpos. (XLSX 1673 kb)

Additional file 3: **File S3.** RNA-Seq data of differentially expressed genes derived from comparison of BL vs. DLBCL, BL vs. DLBCLneg, DLBCLneg vs. DLBCLpos and BL vs. DLBCLpos. (XLSX 13003 kb)

Additional file 4: **Figure S4.** ADGRE5 IHC staining of cell line and patient tissue samples. (A) Burkitt Lymphoma (BL): Cell lines (1–5: Blue-1 Bl-41, BL-2, DG-75, CA-46) and primary tumor tissues (7–14) are manly ADGRE5 positive. (B) Diffuse large B cell lymphoma without *MYC* break (DLBCLneg): Cell lines (1–4: Karpass-422, U2932-R1, HT, WSU-DLCL2) and primary tumor tissues (5–19). (C) DLBCLpos: Cell lines (1–3: Carnaval, U2932-R2, SU-DHL-10) and primary tumor tissues (4–6). DLBCLpos and DLBCLneg are manly negative for ADGRE5. Strong positive staining in some tissue sections results from macrophages or T-cells. (PDF 4769 kb)

## Abbreviations

BL: Burkitt lymphoma
B-NHL: B-cell non-Hodgkin lymphomas
ChIP: Chromatin immunoprecipitation
DLBCL: Diffuse large B-cell lymphoma
DLBCLneg: DLBCL without *MYC* break
DLBCLpos: DLBCL with *MYC* break
EGF-TM7: seven-span transmembrane protein containing epidermal growth factor domains
FDR: False Discovery Rate
GPCR: G protein-coupled receptor
IDR: Irreproducible Discovery Rate
ORI: Origin of Replication
TSS: Transcription start site
