# Supplementary material for: Identification of ADGRE5 as discriminating MYC target between Burkitt lymphoma and diffuse large B-cell lymphoma
Source: BMC Cancer. 2019 Apr 5;19:322. doi: 10.1186/s12885-019-5537-0 (PMC6451309; doi:10.1186/s12885-019-5537-0)
Supplement: Supplementary file 1 — Table S1. Compilation of antibodies used for Chromatin Immunoprecipitation (ChIP), Western Blot (WB) or Immunohistochemistry (IHC) and list of TaqMan Assays used for RT-PCR analysis. *indicated endogenous control. (PDF 42 kb) [file 12885_2019_5537_MOESM1_ESM.pdf]

Primary Antibody					
Target protein	Catalog #	Company	application		
			ChIP	WB	IHC
c-myc	ab32072	Abcam		X	X
c-myc	SC 764 / N-262	SantaCruz	X		
BYSL	ab194961	Abcam		X	
NPM1	TC-161991	Thermo Fisher		X	
Histone H3K4me3	C15410003	Diagenode	X		
Histone 3 *	3638S	Cell Signaling		X	
GAPDH *	2118S	Cell Signaling		X	
beta actin *	Ab6276	Abcam		X	
ADGRE5 (CD97)	HPA013707	Sigma		X	X

Secondary Antibody					
Target	Catalog	Company	ChIP	WB	IHC
Anti-mouse HRP conjugated	P0447	Agilent		X	
Anti-rabbit HRP conjugated	NA934V	GE Healthcare		X	

TAQ-MAN RT-PCR probes		
Target for TaqMan Assay	Assay ID	Company
FARSA	Hs00189494_m1	Thermo Fisher
LARS	Hs00219931_m1	Thermo Fisher
BYSL	custom made	Thermo Fisher
CD30	custom made	Thermo Fisher
TERT	Hs00972650_m1	Thermo Fisher
NPM1	Hs02339479_g1	Thermo Fisher
ADGRE5 (CD97)	Hs00173542_m1	Thermo Fisher
SMAD1	Hs01077084_m1	Thermo Fisher
CDK20	Hs01114921_m1	Thermo Fisher
ZAP70	Hs00896345_m1	Thermo Fisher
GPAM	Hs00326039_m1	Thermo Fisher
MYC	Hs00153408_m1	Thermo Fisher
B2M*	Hs00984230_m1	Thermo Fisher
SDHA*	Hs00417200_m1	Thermo Fisher

S1 Table. Compilation of antibodies used for Chromatin Immunoprecipitation (ChIP), Western Blot (WB) or Immunohistochemistry (IHC) and list of TaqMan Assays used for RT-PCR analysis. *indicated endogenous control.
