# Supplementary material for: Identification of ADGRE5 as discriminating MYC target between Burkitt lymphoma and diffuse large B-cell lymphoma
Source: BMC Cancer. 2019 Apr 5;19:322. doi: 10.1186/s12885-019-5537-0 (PMC6451309; doi:10.1186/s12885-019-5537-0)
Supplement: Supplementary file 4 — Figure S4. ADGRE5 IHC staining of cell line and patient tissue samples. (A) Burkitt Lymphoma (BL): Cell lines (1–5: Blue-1 Bl-41, BL-2, DG-75, CA-46) and primary tumor tissues (7–14) are manly ADGRE5 positive. (B) Diffuse large B cell lymphoma without MYC break (DLBCLneg): Cell lines (1–4: Karpass-422, U2932-R1, HT, WSU-DLCL2) and primary tumor tissues (5–19). (C) DLBCLpos: Cell lines (1–3: Carnaval, U2932-R2, SU-DHL-10) and primary tumor tissues (4–6). DLBCLpos and DLBCLneg are manly negative for ADGRE5. Strong positive staining in some tissue sections results from macrophages or T-cells. (PDF 4769 kb) [file 12885_2019_5537_MOESM4_ESM.pdf]

(A)

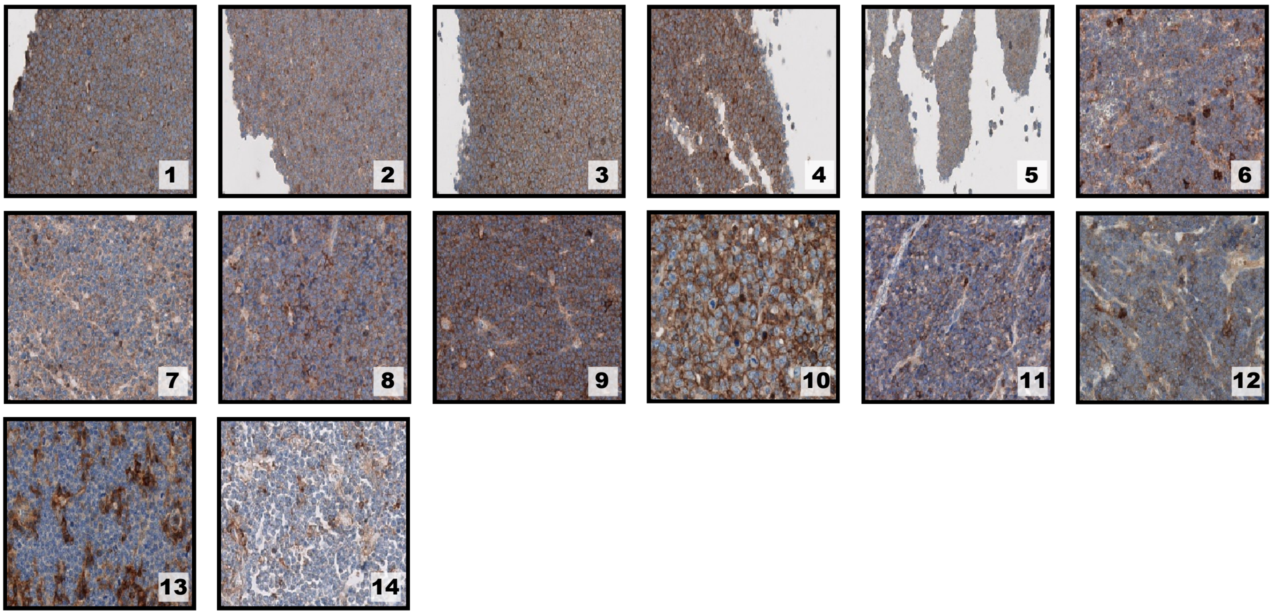

(B)

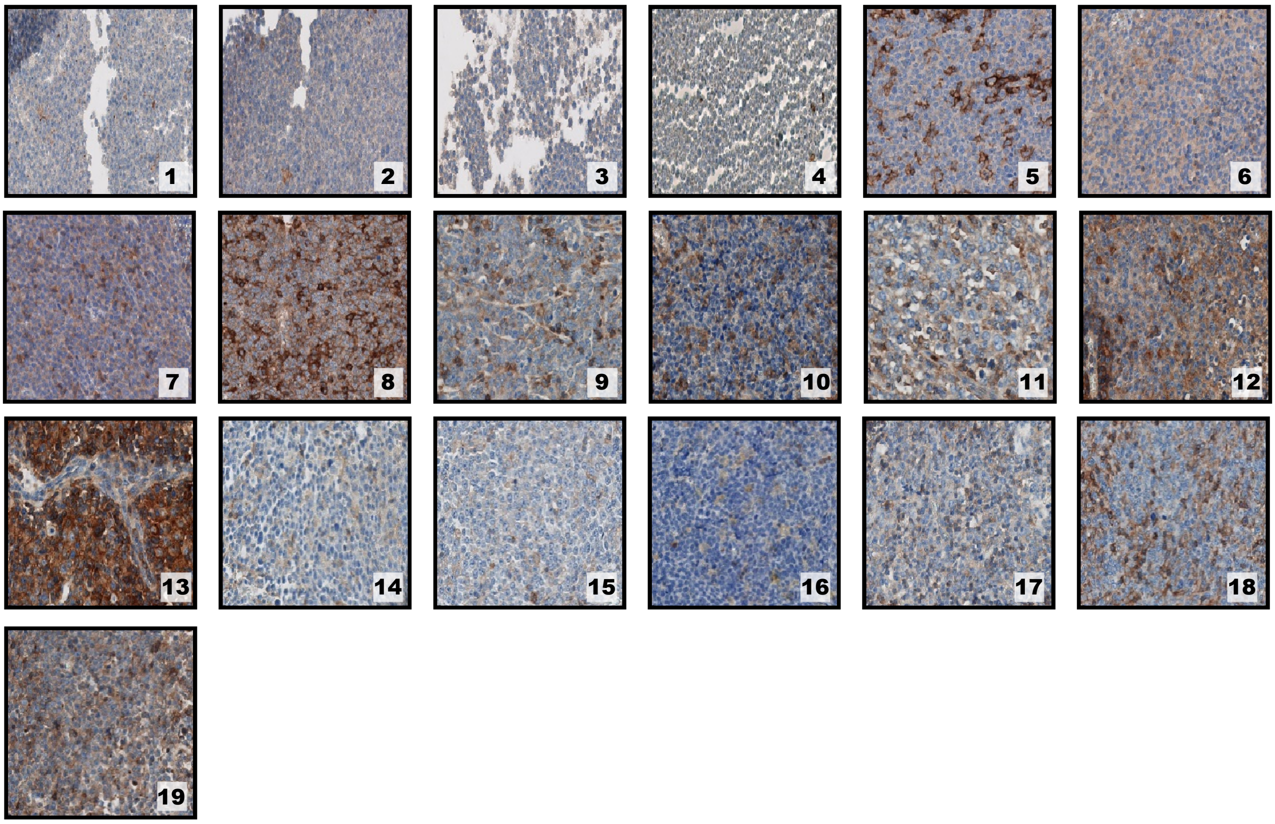

(C)

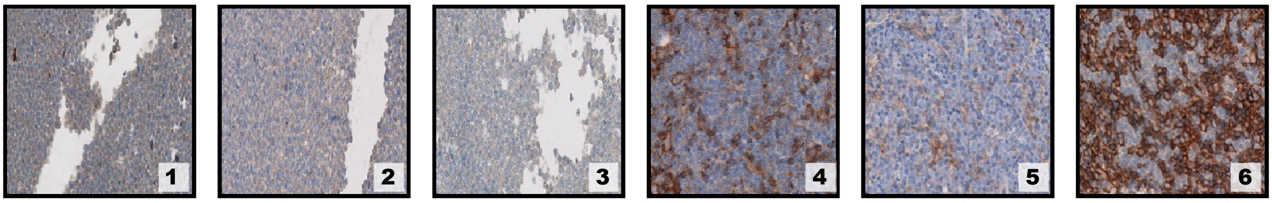

S4 Figure. ADGRE5 IHC staining of cell line and patient tissue samples. (A) Burkitt Lymphoma (BL): Cell lines (1-5: Blue-1 Bl-41, BL-2, DG-75, CA-46) and primary tumor tissues (7-14) are manly ADGRE5 positive. (B) Diffuse large B-cell lymphoma without *MYC* break (DLBCLneg): Cell lines (1-4: Karpass-422, U2932-R1, HT, WSU-DLCL2) and primary tumor tissues (5-19). (C) DLBCLpos: Cell lines (1-3: Carnaval, U2932-R2, SU-DHL-10) and primary tumor tissues (4-6). DLBCLpos and DLBCLneg are manly negative for ADGRE5. Strong positive staining in some tissue sections results from macrophages or T-cells.
